# Involvement of Ferroptosis in Diabetes-Induced Liver Pathology

**DOI:** 10.3390/ijms23169309

**Published:** 2022-08-18

**Authors:** Ana Stancic, Ksenija Velickovic, Milica Markelic, Ilijana Grigorov, Tamara Saksida, Nevena Savic, Milica Vucetic, Vesna Martinovic, Andjelija Ivanovic, Vesna Otasevic

**Affiliations:** 1 Department of Molecular Biology, Institute for Biological Research “Siniša Stanković”, National Institute of Republic of Serbia, University of Belgrade, 11060 Belgrade, Serbia; 2 Department of Cell and Tissue Biology, Faculty of Biology, University of Belgrade, 11060 Belgrade, Serbia; 3 Department of Immunology, Institute for Biological Research “Siniša Stanković”, National Institute of Republic of Serbia, University of Belgrade, 11060 Belgrade, Serbia; 4 Medical Biology Department, Centre Scientifique de Monaco (CSM), 98000 Monaco, Monaco

**Keywords:** ferroptosis, liver, diabetes, oxidative stress, lipid peroxidation, Nrf2

## Abstract

Cell death plays an important role in diabetes-induced liver dysfunction. Ferroptosis is a newly defined regulated cell death caused by iron-dependent lipid peroxidation. Our previous studies have shown that high glucose and streptozotocin (STZ) cause β-cell death through ferroptosis and that ferrostatin-1 (Fer-1), an inhibitor of ferroptosis, improves β-cell viability, islet morphology, and function. This study was aimed to examine in vivo the involvement of ferroptosis in diabetes-related pathological changes in the liver. For this purpose, male C57BL/6 mice, in which diabetes was induced with STZ (40 mg/kg/5 consecutive days), were treated with Fer-1 (1 mg/kg, from day 1–21 day). It was found that in diabetic mice Fer-1 improved serum levels of ALT and triglycerides and decreased liver fibrosis, hepatocytes size, and binucleation. This improvement was due to the Fer-1-induced attenuation of ferroptotic events in the liver of diabetic mice, such as accumulation of pro-oxidative parameters (iron, lipofuscin, 4-HNE), decrease in expression level/activity of antioxidative defense-related molecules (GPX4, Nrf2, xCT, GSH, GCL, HO-1, SOD), and HMGB1 translocation from nucleus into cytosol. We concluded that ferroptosis contributes to diabetes-related pathological changes in the liver and that the targeting of ferroptosis represents a promising approach in the management of diabetes-induced liver injury.

## 1. Introduction

Diabetes mellitus is a chronic metabolic disorder that affects many organs including the liver [1]. Diabetes-related pathological changes in the liver are reflected in the alterations of biochemical serum parameters and morphological and ultrastructural modifications in the organ itself. These alterations in the liver start from fatty degeneration of liver cells and extend to steatohepatitis and periportal fibrosis [2]. Furthermore, diabetes is accompanied by profound alterations in liver size which can be the result of changes in cell number, cell growth, and/or cell death [3,4].

Hepatocellular death is one of the most important contributing factors to diabetes-related liver pathology and progression of liver damage [5]. Therefore, describing the types of cell death and its underlying mechanisms must be in the research focus with the final goal of targeted therapy for this serious diabetic complication. Commonly described types of hepatocellular death in diabetes are apoptosis, autophagy, and necrosis [6,7,8]. Although biochemically and morphologically different, the underlying mechanisms of all those types of cell death involve common components: disturbances in redox/antioxidant and inflammatory status. Both of these develop due to the diabetes-related hyperglycemia and hyperlipidemia and are tightly interconnected. We have recently shown that changes in oxidative state in diabetes induce redox modification of high-mobility group box 1 (HMGB1), an important driver of inflammation signaling. These modifications consequently determine the type of cell death and the cross-talk between apoptosis and autophagy in the liver of diabetic animals [8]. We have also shown that melatonin exerts hepatoprotective effects against pronecrotic events in the liver in experimental diabetes due to its antioxidative properties [6]. These data, along with plenty of others, speak in favor of the benefit of antioxidants supplementation in the diabetic states (reviewed in Johansen et al. [9]). However, the success of the antioxidants’ treatment of diabetes in a clinical study is still missing, suggesting the need for research focusing on the connection between oxidative stress and diabetes-related pathological changes, in the final instance, cell death. In that context, ferroptosis has been examined in the last few years. 

Ferroptosis is a regulated, iron-dependent type of cell death whose backbone makes the cysteine/glutamate exchanger (xCT)-glutathione (GSH)-glutathione peroxidase 4 (GPX4)-membrane hydroperoxide axis [10]. As a cystine/glutamate transporter, xCT provides the cells with cysteine, a building component of GSH that is “incorporated” into this molecule in the reaction catalyzed by the rate-limiting enzyme glutamate-cysteine ligase (GCL). Under homeostatic conditions, oxidative damages occurring at the level of membrane lipids (thus forming lipid peroxides) are promptly removed by the specific membrane-associated isoform of GPX, GPX4 which uses GSH as a reducing power. However, if this antioxidant pathway becomes disturbed, membrane lipid peroxidation propagates throughout the membrane, compromising its integrity and finally resulting in cell death. Starting from the definition of ferroptosis, interest in its involvement in various pathological contexts is growing. To date, it has been reported that ferroptosis is involved in the development of diabetic metabolic complications, including renal injury [11,12,13], cognitive dysfunction [14], osteoporosis [15], and endothelial [16] and retinal [17] injury. We have recently confirmed the implication of ferroptosis in diabetes etiology, as well. Namely, we have found that mimicking the diabetic environment in vitro induced β-cell death through ferroptosis [18]. Moreover, our in vivo pilot study showed that ferrostatin-1 (Fer-1), a commonly used ferroptosis inhibitor, improved islet morphology and functional status along with the decrease in accumulation of lipid peroxides. 

Here, we aimed to reveal the involvement of ferroptosis in diabetes-induced pathological changes in the liver. For that purpose, we examined the effects of Fer-1 on ferroptosis-related parameters in the liver of mice with streptozotocin (STZ)-induced diabetes. Fer-1 is one of the most potent ferroptosis inhibitors that exhibits high efficacy as radical-trapping antioxidants, with a particularly high potency in phospholipid bilayer membranes when compared to other antioxidants [19]. Its metabolic actions in vivo were extensively evidenced, and it is a well-established pharmacological tool for verification of ferroptosis [11,12,18,20,21,22,23,24].

## 2. Results 

### 2.1. Fer-1 Attenuates Diabetes-Induced Liver Damage

As seen in Table 1, a statistically significant difference between the experimental groups was found in the following parameters: body mass, glycaemia, serum ALT and TG level. A Tukey post hoc test revealed that the body mass of diabetic animals was significantly decreased compared to the control group (*p* < 0.05). Body mass of the Fer-1-treated diabetic group was not significantly different from both the control and diabetic group. Furthermore, at day 21 of the experiment, the mean serum glucose level was statistically higher in diabetic animals in comparison to the control (*p* < 0.01). In diabetic animals treated with Fer-1, glycemia was slightly lower than in the DM group but no significant difference was noted when compared to both the DM and Ctrl group. Regarding the hepatogram parameters, a significant elevation in the serum ALT level was found in the diabetic group when compared to the control (*p* < 0.05). The treatment with Fer-1 decreased ALT level compared to the DM group (*p* < 0.05) and restored it towards the control level. TG level was increased in the DM group (*p* < 0.05) in comparison to the control group and was restored with Fer-1 treatment, being lower when compared with the DM group (*p* < 0.05). 

**Figure 1:**
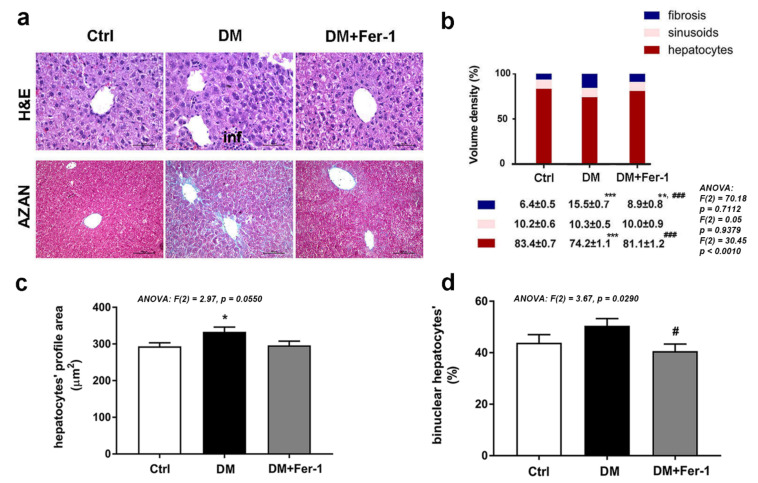
Microscopic analysis of liver tissue of control (Ctrl), diabetic (DM), and diabetic Fer-1-treated (DM + Fer-1) animals. (**a**) Hematoxylin and eosin (H&E) and AZAN trichrome staining; scale bars: H&E—50 µm, AZAN—100 µm, inf—infiltration. (**b**) Volume density of hepatocytes, sinusoids, and fibrosis. (**c**) Hepatocytes profile area. (**d**) Proportion of binuclear hepatocytes. Values presented as means ± SEM. Statistical significance: compared with the Ctrl group (*), * *p* < 0.05, ** *p* < 0.01; *** *p* < 0.001; DM vs. DM + Fer-1 comparison (#), # *p* < 0.05; ### *p* < 0.001.

Histological characteristics of the liver of control and treated mice are presented in Figure 1a. In contrast to the control liver, signs of liver damage including extensive hepatocyte vacuolization, fibrosis, sinusoidal dilation, and immune cell infiltration were observed in diabetic animals. In the DM + Fer-1 group, the above-stated signs of injury were markedly reduced. In AZAN trichrome-stained tissue of diabetic mice, fibrosis was demonstrated as intensive blue staining of collagen around the majority of the central veins and spreading in the perivenous parenchyma. In addition, fibrous expansion of most portal areas with occasional portal-portal and portal-central bridging was observed. Fer-1 treatment reduced liver fibrosis, and collagen depositions were mainly observed in portal areas and in the form of short fibrous septa. These morphological observations were confirmed by stereological analysis since statistical significance among the experimental groups was noted in Vv of hepatic fibrosis (Figure 1b). In comparison with the control, Vv of hepatic fibrosis was increased (*p* < 0.001) in the DM group. By contrast, Fer-1 treatment decreased the fibrotic area compared to diabetic animals (*p* < 0.001), although it still remained increased compared with the control group (*p* < 0.01). There was no difference in Vv of sinusoids between the control, DM, and DM + Fer-1 groups. The analysis of the Vv of hepatocytes revealed statistical significance among the groups and a post hoc test revealed that it decreased in the DM group compared to the control (*p* < 0.001). Fer-1 treatment returned the Vv of hepatocytes to the control level, thus, increasing it in comparison to the DM group (*p* < 0.001). 

Regarding hepatocytes’ size (measured as nucleated profile surface area), a statistically significant difference was noted among the groups, and an increase was found in the DM group compared to the control (*p* < 0.05) (Figure 1c). ANOVA analysis also revealed statistically significant differences between the groups in the ratios of binuclear cells (Figure 1d). Post hoc tests showed no difference between the control and DM group, although an increasing trend of binucleation was noticed in the DM group. By contrast, Fer-1 treatment decreased the size of hepatocytes towards the control level and the proportion of binuclear hepatocytes compared to the DM group (*p* < 0.05). 

### 2.2. Fer-1 Weakens Lipid Peroxidation in Diabetic Liver

**Figure 2:**
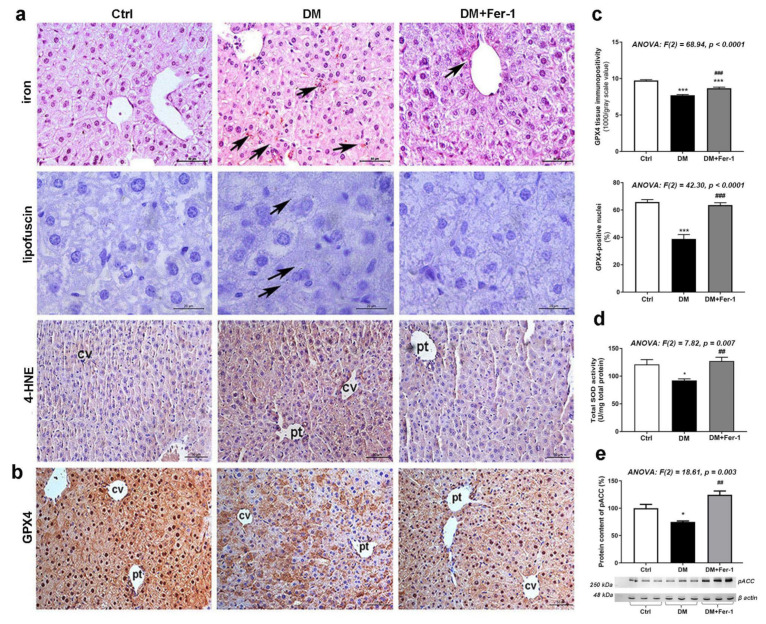
Lipid peroxidation-related and antioxidative parameters in liver tissue in control (Ctrl), diabetic (DM), and diabetic Fer-1-treated (DM + Fer-1) animals. (**a**) DAB-enhanced Pearls’ iron staining demonstration of ferrous ions accumulation (arrows); Sudan III stain-detected lipofuscin (arrows) and immunohistochemical detection of 4-HNE; scale bars: iron and 4-HNE—50 µm, lipofuscin—20 µm. (**b**) Immunohistochemical detection of GPX4; scale bars: 50 µm and (**c**) quantification of tissue and nuclear GPX4 immunopositivity; cv—centrilobular vein, pv—portal vein. (**d**) Total SOD activity. (**e**) Protein content of pACC; β-actin serves as a protein-loading control; blots represent three independent experiments. Values presented as means ± SEM. Statistical significance: compared with the Ctrl group (*), * *p* < 0.05, *** *p* < 0.001; DM vs. DM + Fer-1 comparison (#), ## *p* < 0.01; ### *p* < 0.001.

As expected, we have demonstrated signs of increased hepatic lipid peroxidation including the increased iron deposition, and the accumulation of lipofuscin and 4-HNE, the second product of lipid peroxidation (Figure 2a). While immunopositivity for 4-HNE was diffuse, homogeneously distributed among hepatocytes in the control tissue, microscopic observation revealed numerous concentrated areas of 4-HNE accumulation in the periportal and pericentral regions of the livers of the DM group. A similar pattern of 4-HNE localization was noticed in the DM + Fer-1 group, however, only some areas with increased 4-HNE positivity were noticed throughout the hepatic lobules. On the contrary, Fer-1 decreased the levels of these parameters towards control levels, including the accumulation of iron, lipofuscin, and 4-HNE. GPX4 immunopositivity showed an opposite pattern of changes compared to those parameters, reflecting lipid peroxide accumulation (Figure 2b,c). Namely, a significant difference among the experimental groups was noted in GPX4 expression at the tissue level, and post hoc tests revealed that its expression was decreased in diabetes (*p* < 0.001), while Fer-1 treatment alleviated this effect of DM, although it still remained below the control level (*p* < 0.001) (Figure 2c). A similar pattern was found after the counting of GPX4-positive nuclei ratio (Figure 2c) where significant difference between the groups was also noted, while the post hoc test revealed a decrease in the DM group in comparison to the control (*p* < 0.001) and an increase in the DM + Fer-1 group when compared to the DM group (*p* < 0.001). Additionally, statistical significance in the activity of total SOD (Figure 2d) and level of the pACC (Figure 2e) were also demonstrated and post hoc tests revealed decreases in the DM group compared to the control (*p* < 0.05), as well as the increase for both parameters in DM + Fer-1 group compared to the DM group (*p* < 0.01). 

### 2.3. Fer-1 Improves Diabetes-Induced Attenuation of GSH-Related Antioxidant Defense in the Liver

**Figure 3:**
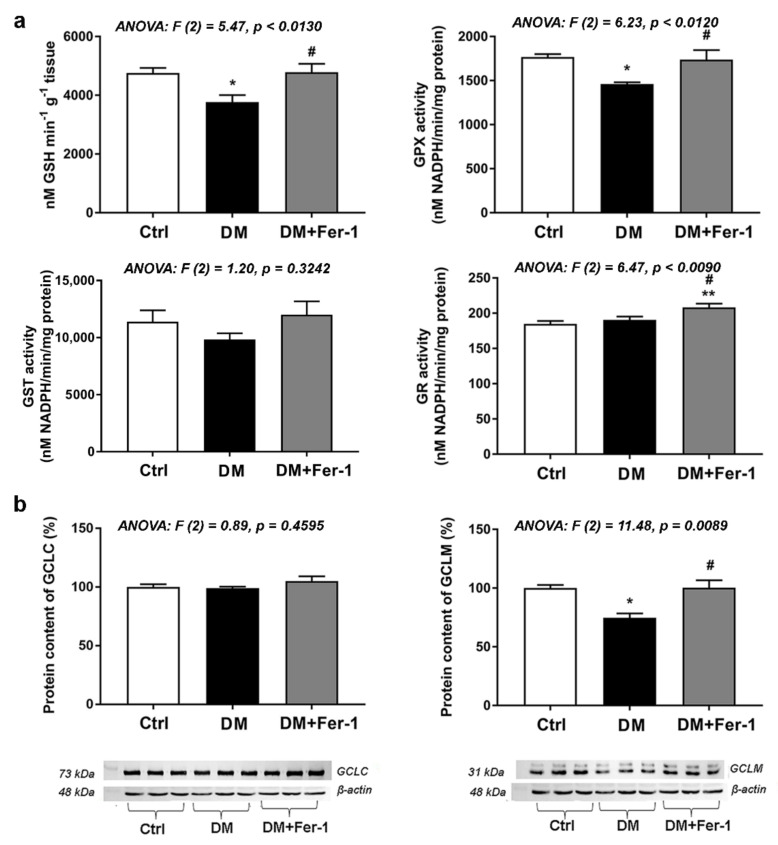
GSH and GSH-related enzymes in the liver of control (Ctrl), diabetic (DM), and diabetic Fer-1-treated (DM + Fer-1) animals. (**a**) Content of GSH and activities of GPX, GST, and GR. (**b**) Protein content of GCLC and GCLM; β-actin serves as a protein-loading control; blots represent three independent experiments. Values presented as means ± SEM. Statistical significance: compared with the Ctrl group (*), * *p* < 0.05, ** *p* < 0.01; DM vs. DM + Fer-1 comparison (#), # *p* < 0.05.

Changes in the GSH content and activities of GSH-related antioxidative enzymes are summarized in Figure 3a. A significant difference among the groups in the content of GSH and activity of GPX was found in the liver (Figure 3a), being decreased in the DM group when compared to the control (*p* < 0.05). Both GSH content and GPX activity in the DM + Fer-1 group returned to the control level and increased significantly compared to the DM group (Figure 3a). Although there were no significant changes in GST activity between the groups, there was a decreasing trend in the DM group compared to control, which disappeared in the DM + Fer-1 group. GR activity in the liver was also significantly different between the groups, and a post hoc test revealed that Fer-1 treatment of diabetic animals increased activity of GR compared with both the control and diabetic group (*p* < 0.01 and *p* < 0.05, respectively).

Figure 3b shows the protein content of GCL, i.e., its catalytic subunit (GCLC) and its modifier (GCLM) subunit. While there was no significant difference in GCLC protein content, GCLM protein level was significantly different between the groups. Namely, it was decreased in the diabetic group compared to the control (*p* < 0.05), while Fer-1 treatment returned it to the control level, thus, increasing it in comparison to the DM group (*p* < 0.05). 

### 2.4. Fer-1 Re-Establishes Diabetes-Induced Disturbances in Hepatic Nrf2 Signaling 

**Figure 4:**
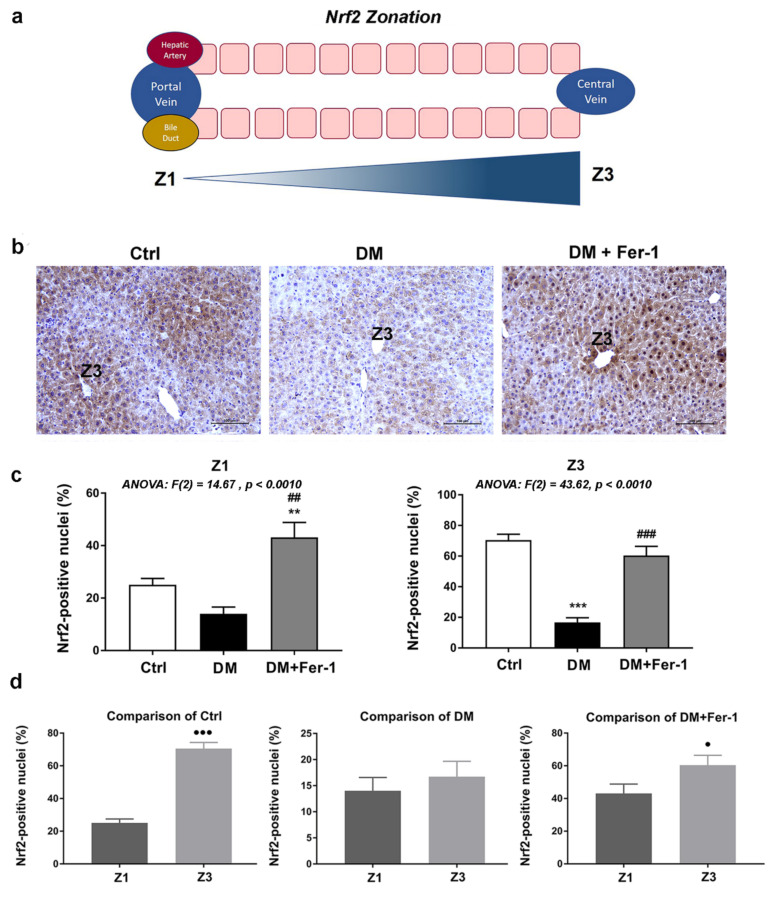
Immunohistochemistry of Nrf2 in liver tissue of control (Ctrl), diabetic (DM), and diabetic Fer-1-treated (DM + Fer-1) animals. (**a**) Schematic overview of structural zonation of the liver. (**b**) Immunohistochemical detection of Nrf2 expression across the liver zones; scale bars—50 µm. (**c**) Proportion of Nrf2 positive nuclei between the groups in Z1 and Z3. (**d**) Proportion of Nrf2 positive nuclei between the zones in control, DM, and DM + Fer-1 groups. Values presented as means ± SEM. Statistical significance: compared with the Ctrl group (*), ** *p* < 0.01; *** *p* < 0.001; DM vs. DM + Fer-1 comparison (#), ## *p* < 0.01; ### *p* < 0.001. Z1 vs. Z3 comparison (^●^), ^●^ *p* < 0.05, ^●●●^ *p* < 0.001.

Strong Nrf2 cytoplasmic and nuclear immunopositivity of hepatocytes was noted in the liver of control animals (Figure 4). In addition, a lobular zonation in its expression was noted (Figure 4d), since the strongest immunopositivity of nuclei was demonstrated in the pericentral (Z3) zone when compared to the periportal (Z1) zone of the control group (*p* < 0.001). This zonation difference declines in the DM group, while Fer-1 treatment returns it toward control level (*p* < 0.05). Comparison of nuclear Nrf2 immunopositivity in both Z1 and Z3 revealed statistically significant differences among the groups (Figure 4c). Nuclear Nrf2 immunopositivity of hepatocytes decreased in the lobular zones of the DM group. This is especially notable in the Z3 lobular zone where statistical significance in comparison to the control was noted (*p* < 0.001). Fer-1 treatment of diabetic animals returned Nrf2 nuclear immunopositivity to control level (in Z3, *p* < 0.001 in comparison to DM group) and even increased it (in Z1) above control and DM level (*p* < 0.01).

**Figure 5:**
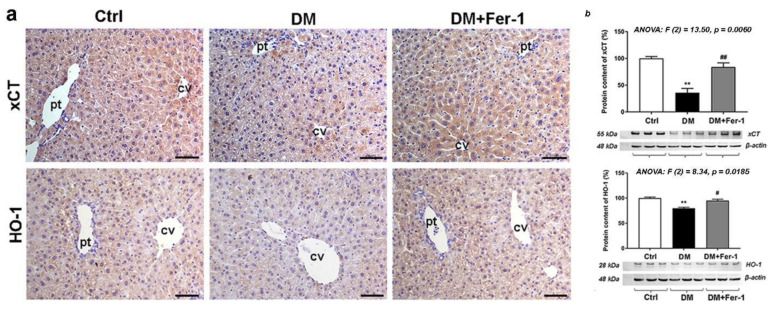
Detection of xCT and HO-1 (**a**) immunohistochemical localization and (**b**) protein expression in the liver tissue of control (Ctrl), diabetic (DM), and diabetic Fer-1-treated (DM + Fer-1) animals. Scale bars: 50 μm; cv—centrilobular vein, pv—portal vein. Values presented as means ± SEM. Statistical significance: compared with the Ctrl group (*), ** *p* < 0.01. DM vs. DM + Fer-1 comparison (#), # *p* < 0.05, ## *p* < 0.01.

In addition to diabetes-related changes in the expression of some ferroptosis-related downstream targets presented above (GPX4 and SOD in Figure 2 and GCLM in Figure 3), changes in the expression of xCT and HO-1 are presented at Figure 5. Compared to the control, the lowest xCT and HO-1 immunopositivity was found in the hepatocytes of diabetic animals (Figure 5a), which were restored in the DM + Fer-1 group. These results were confirmed by Western blot (Figure 5b) since significant differences were demonstrated for both the xCT and HO-1 protein content among the experimental groups. Post hoc tests revealed the significant decrease in xCT (*p* < 0.01) and HO-1 (*p* < 0.01) in the DM group and significant increase after Fer-1 treatment (*p* < 0.01 and *p* < 0.05, respectively, in comparison to the DM group). Additionally, lobular zonation of xCT immunopositivity was noted in both the control and DM + Fer-1 groups, since the highest immunoreactivity was observed around the portal and the centrilobular vein, i.e., in the periportal (Z1) and pericentral area (Z3). 

### 2.5. Fer-1 Abrogates Diabetes-Induced Activation of HMGB1 and Increase in Inflammatory Cytokines

**Figure 6:**
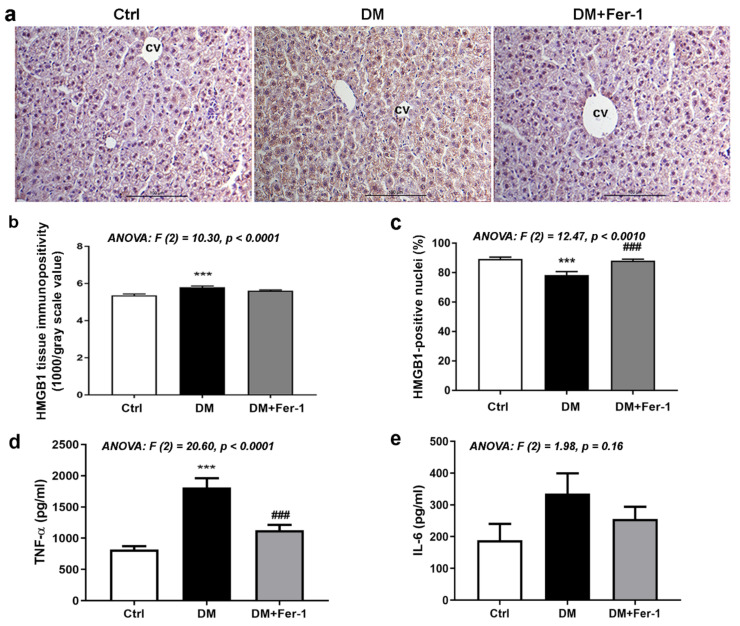
HMGB1, TNF-α, and IL-6 in liver tissue of control (Ctrl), diabetic (DM), and diabetic Fer-1-treated (DM + Fer-1) animals. (**a**) Immunohistochemical detection of HMGB1; scale bars—50 µm. (**b**) Quantification of tissue and (**c**) nuclear immunopositivity of HMGB1. Protein level of TNF-α (**d**) and IL-6 (**e**) measured by ELISA. Values presented as means ± SEM. Statistical significance: compared with the Ctrl group (*), *** *p* < 0.001. DM vs. DM + Fer-1 comparison (#), ### *p* < 0.001.

Figure 6 presents the results of immunohistochemical detection of HMGB1 (a, b, and c) and analysis of protein content of TNF-α (d) and IL-6 (e). As it has been observed, HMGB1 is mostly localized in the nuclei of hepatocytes in the liver of control animals. Diabetes led to an increase in HMGB1 tissue immunopositivity since strong/moderate HMGB1 immunopositivity is detectable in the cytoplasm of many hepatocytes. However, nuclear HMGB1 positivity of these cells declined in the DM group. Fer-1 treatment of diabetic mice re-established nuclear translocation of this protein in hepatocytes and decreased overall tissue and nuclear HMGB1 immunopositivity. Microscopic observations were confirmed quantitatively since statistically significant differences in overall tissue and nuclear HMGB1 immunopositivity were noted among the experimental groups (Figure 6b,c). Compared to the control, there was a statistically significant increase in liver immunopositivity of the DM animals, while the percentage of HMGB1 positive nuclei decreased (*p* < 0.001, both). Fer-1 treatment restored the percentage of HMGB1 positive nuclei to the control level, thus increasing it compared to the diabetic group (*p* < 0.001). Protein content of TNF-α (Figure 6d) was significantly increased in the DM group compared to the control (*p* < 0.001), while Fer-1 treatment restored it to the control and decreased it compared to the DM group (*p* < 0.001). Although the changes in the amount of IL-6 protein are in the same direction as TNF-α, they only showed a trend of increase in the DM and a trend of decrease in the diabetic group treated with Fer-1 (Figure 6e).

## 3. Discussion

Cell death assumes a central role in the etiology of most liver pathologies, including those that are diabetes-related [5]. We described, here, the ferroptotic phenotype in diabetic liver. All observed ferroptotic events: (i) increased accumulation of pro-oxidative (such as iron, lipofuscin, and 4-HNE) and pro-inflammatory (HMGB1) markers and (ii) decrease in antioxidative defense-related molecules (Nrf2, xCT, GSH, GPX4, GCL, HO-1, SOD) in the liver of diabetic animals were diminished after the treatment with ferroptosis inhibitor, Fer-1. Such beneficial effects of Fer-1 were reflected in the normalization of diabetes-induced alterations in the liver metabolism (ALT and TG) and structure (less fibrosis, unaltered hepatocytes size). Those changes induced by Fer-1 in the liver of diabetic mice were discussed below.

The first indicators of the benefit of Fer-1 treatment on the diabetic liver were recoveries of biochemical markers and histology/morphology of the liver itself. The fact that Fer-1 decreased the diabetes-induced increase in the level of ALT and TG suggests that ferroptosis indeed contributes to diabetes-related metabolic/functional disturbances in the liver. These changes are further supported by histological analyses. Extensive deposition of collagen inside the extracellular matrix (fibrosis), a common indicator of liver damage in diabetic conditions [25,26,27], was reversed by Fer-1 treatment. Along with this, Fer-1 normalized hepatocytes size, morphology, and decreased the number of binuclear cells in the diabetic liver, a phenomenon which is indicative of liver regeneration [28]. Enlarged hepatocyte size in the diabetic liver could be a consequence of hepatocytes’ ballooning and/or polyploidization which are both indicative of liver cell degeneration [26,29] observed in the conditions including iron and copper overload [30] and oxidative stress [31].

Oxidative stress is the crossing link between diabetes-induced systemic metabolic disturbances (hyperglycemia, hyperlipidemia, inflammation) and pathological changes, functional and morphological, in many tissues/organs, including liver [32]. An increased level in the markers of oxidative stress, including the markers of lipid peroxidation has been repeatedly shown in the diabetic liver [32,33] and is recognized as a causative factor of hepatocellular death [34]. The results of the present study, showing that Fer-1 reduced the diabetes-induced increase in the level of 4-HNE, a commonly studied product of lipid peroxidation, put the process of accumulation of lipid peroxides in the diabetic liver in the context of ferroptosis. Along with this, Fer-1 abolished the decrease in total SOD activity in diabetes and returned it to the control level. As is already known, an increased oxidation of poly-unsaturated fatty acids containing phospholipids (PUFAs) and accumulation of lipid peroxides represents the main event in ferroptosis [10]. It results from a disbalance in the production of lipid peroxides and their removal. Nonenzymatic production of lipid peroxides goes through the Fenton reaction [35] involving the interaction of ferrous iron (Fe^2+^) with H_2_O_2_. The diabetic state is characterized by the intracellular iron deposition that has been correlated with the diabetes-induced pathological changes in many tissues, including liver [36]. Since iron represents the key catalyst of the Fenton reaction and consequent lipid peroxidation, it is not surprising that diabetes-related iron deposition may lead to ferroptosis. Our study confirms that hypothesis since Fer-1 treatment decreased the diabetes-induced increase in iron content in the liver. One more parameter speaks in favor of iron-dependent oxidative damage in diabetic liver. We have found that lipofuscin showed a similar pattern of changes as the iron content and that its formation could be related to ferroptosis in the diabetic liver, as its accumulation in diabetes was diminished by Fer-1. This is in line with our recently published data where we characterized the relationship between the lipofuscin accumulation and ferroptosis in β-cells under the diabetogenic conditions in vitro [18].

Along with those parameters of lipid peroxidation, we also found that Fer-1 induced restitution of diabetes-induced decrease in the level of pACC. ACC is a central enzyme involved in fatty acid biosynthesis, including long chain PUFAs and, thus, plays a context-dependent role in promoting ferroptosis. It has been shown that the inhibition of ACC blocks both erastin- and ras-selective lethal small molecule 3 (RSL3)-induced ferroptosis in mouse embryonic fibroblasts [37]. Inhibition of ACC is mediated by AMPK-induced phosphorylation that leads to suppression of the de novo lipogenesis pathway and, thus, inhibition of ferroptosis [38]. Targeted inhibition of ACC has been suggested as a therapeutic strategy in several liver disorders [39]. Our results suggest that Fer-1 antiferroptotic effects in the liver of diabetic animals involve such mechanisms.

On the other hand, the removal of lipid peroxides is catalyzed by a specific membrane-associated isoform of GPX, GPX4. GPX4 has been considered a primary enzymatic defense mechanism against reactive oxygen species (ROS)-mediated membrane peroxides and consequently against ferroptosis, due to its strong association with membranes and its close proximity to phospholipid peroxide substrates [40,41,42]. Specifically for the liver, Conrad’s group [43] revealed GPX4 as critical for hepatocyte survival and proper liver function, and that vitamin E can compensate for its loss by protecting cells against deleterious lipid peroxidation and cell death in various pathological contexts. We have found, here, that Fer-1 treatment not only compensated for the decrease in GPX4 expression in diabetic liver but also improved the GPX4-mediated lipid peroxides removal. Namely, we have found that Fer-1 restored the GPX4 hepatic expression that was decreased in diabetes and abolished the diabetes-induced decrease in total GPX activity. Interestingly, our immunohistochemical analysis additionally showed strong nuclear localization of GPX4 in the control group, which was decreased in diabetes and normalized by Fer-1 treatment. GPX4 has been reported to be localized in nuclear, mitochondrial, and/or cytoplasmic cellular compartments [44]. Nuclear GPX4 can inhibit the activation of 5-lipoxygenase, thus, suppressing the enzymatic production of lipid peroxides in this compartment [45]. Additionally, GPX4 in the nucleus may protect DNA integrity by preventing lipid peroxidation-induced DNA damage, e.g., etheno-nucleotide adducts, and via direct repair of oxidatively damaged DNA adducts such as thymine hydroperoxide [46,47]. The nuclear localization of GPX4, presented in this study, supports the significance of this aspect of its action in the protection of hepatocytes, at least against diabetogenic damage.

In addition to GPX4, Fer-1 restored the activity of GST which showed a decreasing trend in diabetes. It is well-known that GST is one of the three main 4-HNE-metabolizing enzymes, among aldehyde dehydrogenase and alcohol dehydrogenase [48]. So, the protective effects of Fer-1 in the diabetic liver should come either from its direct lipid peroxide scavenging activity or from its indirect effect on the stimulation of the enzymatic removal of lipid peroxides (GPX4) or of their secondary products, such as 4-HNE (GST).

The main regulator of redox homeostasis and cellular defense against injury is Nrf2 [49,50]. After its activation, which implies its nuclear translocation, Nrf2 endows cells with protective mechanisms against oxidative stress via transcriptional activation of a series of detoxification genes, such as phase II detoxification enzymes HO-1 and GST and antioxidant-related molecules (xCT, GCL, GPX, SOD) [51,52,53]. Impaired Nrf2 signaling in diabetic conditions has been reported extensively [8,18,54,55]. We have recently reported that impaired Nrf2 signaling led to β-cell ferroptosis in diabetic conditions in vitro and in vivo [18]. Additionally, we described that the impaired Nrf2 signaling is an important part of the overall inflammatory and oxidative state in the diabetic liver [36]. The results from the present study suggest that compromised Nrf2 signaling is also an important part of hepatocytes’ ferroptotic phenotype in diabetes since Fer-1 treatment restored the diabetes-induced decrease in Nrf2 nuclear immunopositivity i.e., its inactivation.

Moreover, here, we noticed the differential expression of Nrf2 across the liver zones. Namely, the highest level of Nrf2 expression noticed in the pericentral zones in both the control and Fer-1-treated groups could be a consequence of more hypoxic and nutrient-sparse conditions in this tissue area [56]. The protective role of Nrf2 in hypoxic conditions is already recognized in several cell types, particularly liver and kidney cells [57,58]. It is known that metabolic liver zonation is important for normal liver function, as well as that liver cells differentially express zone-specific genes [59]. In addition, it has been shown that some liver diseases have zonal preferences, while others disrupt physiological liver zonation, which may contribute to disease progression [60]. Taking this into account along with disruption of the liver zonal pattern of Nrf2 expression in the diabetic group and its restitution by Fer-1, it might be supposed that spatial expression of Nrf2 is an important prerequisite for the normal liver function and that this aspect of understanding the role of Nrf2 in diabetes pathology must be further taken into consideration.

Nrf2 is the key regulator of the molecules that make the core of ferroptotic signaling—xCT/GSH/GPX4. We observed that, along with the reduced Nrf2 activation in diabetes, there was also a decrease in the content of GSH and protein expression of xCT, GCLM, and GPX4 (seen as a decrease in overall tissue immunopositivity and protein content). GCLM represents the modifier subunit of GCL, a rate-limiting enzyme for GSH synthesis. GCLM binds to the catalytic subunit (GCLC) and determines the enzyme activity and, thus, GSH level [61]. Results from mice lacking GCLM clearly demonstrate that it has a limiting role in maintaining cellular GSH levels in many tissues, including liver [62,63]. Our results suggest that GCLM could be the limiting factor for GSH synthesis in the diabetic liver since the expression of this subunit, but not the catalytic, decreased in diabetes. The restitution of Nrf2 hepatic activity by the Fer-1 treatment is accompanied by the recovery of the examined ferroptosis-related Nrf2 targets in the liver, thus supporting our hypothesis about the importance of Nrf2 signaling in the determination of hepatocytes’ ferroptotic phenotype in diabetes.

Among those parameters tightly related to ferroptosis, we have examined one more target of Nrf2 signaling, HO-1. The Nrf2/HO-1 signaling pathway was recently described to be involved in the antagonizing oxidative stress-related damage in multiple organs and various pathological injuries [64]. There is also growing data that put the activation of the Nrf2/HO-1 pathway in the antiferroptotic context in various tissues [15,65]. The present study suggests the role of this pathway in the protection of the liver from ferroptosis in diabetic conditions since HO-1 protein expression and immunopositivity have a similar pattern of changes as Nrf2 in the examined conditions (decreased in diabetes and restituted after the Fer-1 treatment).

Along with these pro-oxidative events, pro-inflammatory components play an important role in the pathology of the diabetic liver. In that context, HMGB1 has been recognized as important in controlling the fate of liver cells as well as in determining the type of cell death (apoptosis or autophagy) in the progression of liver damage in diabetes [8]. Switching from its physiological function in the nucleus [66] to its pathological impact (inflammation and cell death) in various pathological conditions, including diabetes, involves its translocation from the nucleus to the cytoplasm, and further to extracellular space [67]. We and others have shown recently that the protein level of HMGB1 is significantly increased in the serum and liver of both diabetic animals and patients [6,68]. In the present study, we observed that Fer-1 treatment reduced diabetes-induced nuclear-to-cytoplasmic translocation of HMGB1, and it increased overall expression. Such inhibition of diabetes-driven activation of HMGB1 by Fer-1 coincided with the reduction of all the examined ferroptosis events in the liver, strongly suggesting the involvement of HMGB1 in the regulation of diabetes-induced liver ferroptosis. Recently, HMGB1 emerged as a novel regulator of ferroptosis in several pathological contexts [69,70,71]. According to Ye et al. [71], HMGB1 is directly involved in the positive regulation and maintenance of ferroptosis in erastin-treated HL-60/NRAS Q61L cells, possibly through regulation of iron-mediated lipid ROS production. Findings from the present study highlight for the first time the involvement of HMGB1 in the regulation of ferroptosis in the liver of diabetic animals and the potential of HMGB1 inhibition in the context of liver protection from this form of cell death in diabetes.

It seems likely that ferroptosis in the liver of diabetic animals is accompanied also with the increase in level of the inflammatory cytokines (primarily TNF-α), since Fer-1 treatment abolishes the diabetes-induced increase in the level of this commonly studied marker of inflammation in the liver [72]. In addition to HMGB1, these results confirm the connection between inflammatory events and ferroptosis in diabetic liver.

## 4. Materials and Methods

### 4.1. Experimental Design

Male C57BL/6 mice were housed with unlimited access to standard chow and tap water at the animal facility at the Institute for Biological Research “Sinisa Stankovic”. All experimental procedures were approved by the Ethic Committee at the Institute for Biological Research “Sinisa Stankovic” (App. No 323-37-11487/2021-05) in accordance with the Directive 2010/63/EU. The 8–10 weeks old male C57BL/6 mice were divided into three groups (*n* = 8): diabetic (DM), diabetic + Fer-1-treated (DM + Fer-1), and control (Ctrl). Diabetes was induced using multiple low doses of STZ (40 mg/kg bm; S0130, Sigma-Aldrich, St. Louis, MI, USA), which were given intraperitoneally for 5 consecutive days (day 1–5) as performed previously [18]. Fer-1, 1 mg/kg bm (SML 0583, Sigma-Aldrich, St. Louis, MI, USA), was dissolved in dimethyl sulfoxide (DMSO, D8418, Sigma-Aldrich, St. Louis, MI, USA) first and then diluted in phosphate-buffered saline (PBS), and administered intraperitoneally, starting from the first dose of STZ for 21 days in total (from day 1–21). To avoid possible interference, the injections of STZ and Fer-1 were given 3 h apart. The control group received the diluents in equal volume. On day 22, 24 h after the last Fer-1 dose, all animals were euthanized between 9:00 and 9:30 AM; blood and liver were collected and routinely prepared for biochemical, microscopic, immunoblot and/or spectrophotometric analysis.

### 4.2. Biochemical Serum Analysis 

Immediately after the blood collection, the animals were euthanized, and the liver perfused with saline. Serum was prepared [73] and stored at −80 °C until analysis. Serum glucose level was determined commercially in the biochemical laboratory Beograd (Belgrade, Serbia). Hepatogram, i.e., content of alanine transaminase (ALT) and triglycerides (TG) in serum were measured spectrophotometrically (Shimadzu UV-160 spectrophotometer, Kyoto, Japan) using an ALT and TG colorimetric assay kit according to the manufacturer’s instructions (ALT-250; TRG-200, respectively, Bioanalytica, Belgrade, Serbia).

### 4.3. Microscopic Examination 

Immediately after dissection, samples of the liver’s median lobe were cut and fixed in 10% formaldehyde at 4 °C overnight, processed routinely for embedding in paraffin blocks and cut into 5 μm thin sections for microscopic analyses. 

#### 4.3.1. Histological, Morphometric, and Stereological Analyses

For routine histological analysis, as well as for the morphometric and stereological analyses (ratio of mono/binuclear hepatocytes, hepatocyte’s nucleated profile area) liver sections were routinely stained with hematoxylin and eosin. Furthermore, AZAN trichrome staining of the liver was performed to determine the volume density (Vv) of the major hepatic tissue components (sinusoids, hepatocytes, and fibrotic area) [74]. The stage of fibrosis, a reflection of the extensive collagen deposition, is visible as increased blue staining of the interstitium [75]. The sections were examined with the Leica DMLB microscope (Leica Microsystems, Wetzlar, Germany). For all these analyses, 50 randomly selected micrographs per group were used, with an objective lens magnification of ×40.

Volume densities (Vv) were determined using Vv = *P_x_*/*P_t_*, where *P*_x_ is the number of points hitting the structure and *P*_t_ is the number of total points hitting the tissue [76]. The Vv values were expressed as percentage fractions.

#### 4.3.2. Iron Staining 

The intracellular presence of nonheme iron (Fe(III)) was evaluated by 3,3′-diaminobenzidine (DAB)-enhanced Pearl’s iron staining [77]. Briefly, sections were deparaffinized and dehydrated routinely and incubated in Prussian blue solution for 8 h. After washing, tissue was incubated in DAB chromogen solution (0.06% H_2_O_2_ in 0.05% DAB in PBS for 10 min), while counterstaining was carried out with hematoxylin. As a positive control, sections of the spleen were used. The sections were washed, rehydrated, mounted in dibutyl phthalate polystyrene xylene (DPX) (06522, Sigma-Aldrich, St. Louis, MI, USA), and examined with a Leica DMLB microscope. The cells exhibiting brown particles were considered iron positive.

#### 4.3.3. Lipofuscin Detection 

After deparaffinization and rehydration, liver sections were stained with 60% Sudan III dye solution in water (0.5 g in 100 mL of 99% isopropanol). After 15 min, slides were washed, counterstained with Mayer’s hematoxylin, mounted in DPX, and examined with a Leica DMLB microscope. The cells exhibiting dark intracellular particles were considered lipofuscin-positive, and the percentage of positive cells was determined within 50 randomly selected micrographs per experimental group. 

#### 4.3.4. Immunohistochemistry

To detect the expression and localization of NF-E2-related factor 2 (Nrf2), GPX4, xCT, heme oxygenase 1 (HO-1), and HMGB1, as well the formation of 4-hydroxynonenal (4-HNE)-protein adducts in the liver, immunohistochemistry was performed. The 5 μm thick liver sections were deparaffinized by xylene and rehydrated in graded ethanol. Blocking of endogenous peroxidase activity, antigen retrieval, and blocking of nonspecific binding with normal goat serum (1:10, K 3408, Dako liquid DAB+ Substrate Chromogen substrate System, Carpinteria, CA, USA) were performed as we had implemented previously. Samples were then incubated overnight at 4 °C with following rabbit primary antibodies: anti-4-HNE (ab46545, 1:500), anti-Nrf2 (ab31163, 1:100), anti-GPX4 (ab125066, 1:100), anti-HO-1 (ab13243, 1 μg/mL), anti-HMGB1 (ab18256, 1 μg/mL), all purchased from Abcam (Cambridge, UK) and goat anti-xCT (sc79360, 1:100), purchased from Santa Cruz Biotechnology (Santa Cruz, TX, USA). After rinsing with PBS, the sections were incubated with appropriate secondary antibodies: goat antirabbit (ab97051, 1:1000, Abcam) and donkey antigoat (sc-2020, 1:4000, Santa Cruz Biotechnology) for 1 h at room temperature. The final reaction product was visualized with DAB chromogen solution (K 3408, Dako liquid DAB+ Substrate Chromogen substrate System, Carpinteria, CA, USA). After counterstaining with hematoxylin, slides were mounted in DPX and examined with a light microscope (Leica Microsystems). To quantify the HMGB1 and GPX4 tissue immunopositivity, approximately 20 fields from micrographs at 40x objective magnification were analyzed (two animals per group). Micrographs were analyzed in Image J software (National Institutes of Health, Bethesda, MA, USA) by the Color Deconvolution2 plugin in which images were split on hematoxylin and DAB channels. DAB channel images were analyzed by random selection of fields falling on the tissue (with the omission of large vessels). Obtained grayscale values (from 0 = black—the strongest signal, to 255 = white—no signal) were divided by 1000 to get the values that will be directly proportional to immunopositivity strength. To quantify the percentage of HMGB1- and GPX4-positive nuclei, approximately 20 micrographs at 20× objective magnification were analyzed (two animals per group) at Image J software (National Institutes of Health) The total number of nuclei was used to calculate the percentage of hepatocytes exhibiting nuclear staining. Quantification of nuclear Nrf2 staining was determined from 50 micrographs at 40× magnification (25 from pericentral, and 25 from periportal area, two animals per group). The total number of nuclei was used to calculate the percentage of hepatocytes exhibiting nuclear staining.

### 4.4. Analysis of GSH Content and Activities of Antioxidative Defense Enzymes 

For the examinations of the antioxidative defense system, the liver tissue was dissected out and thoroughly rinsed with saline to remove traces of blood. To measure the activities of antioxidative enzymes in the liver, a 10% homogenate of the liver, prepared in sucrose buffer (0.25 M sucrose, 0.1 mM EDTA, and 50 mM Tris-HCl pH 7.4), was used. The activity of GPX was determined spectrophotometrically with t-butyl hydroperoxide as a substrate [78] and expressed in nmol of reduced NADPH min^−1^ mg^−1^ protein. Glutathione S-transferase (GST) activity was measured by the method of Habig et al. [79] and expressed in nmol GSH min^−1^ mg^−1^ protein. Activity of GSH reductase (GR) was assayed by the method of Glatzle et al. [80] and expressed as nmol NADPH min^−1^ mg^−1^ protein. The content of GSH was examined in the tissue after deproteinization with 10% sulfosalicylic acid. Total GSH was measured by the enzyme-recycling assay of Griffith [81] and expressed in nmol GSH min^−1^ g^−1^ tissue. Total superoxide dismutase (SOD) activity was assayed using the method described by Misra and Fridovich [82], but at 26 °C and expressed in units mg^−1^ of protein. SOD units were defined as the amount of the enzyme inhibiting epinephrine auto-oxidation under the appropriate reaction conditions. 

### 4.5. SDS-Polyacrylamide Gel Electrophoresis (PAGE) and Western Blot Analysis

For SDS-PAGE and Western blot analysis, a 10% homogenate of liver, prepared in sucrose buffer containing protease and phosphatase inhibitors (Protease-Inhibitor-Mix G, #39101, Serva Electrophoresis, Heidelberg, Germany), was used as described previously [18]. Protein content in the samples was estimated by the method of Lowry et al. [83]. Ten μg of total protein extracts was separated by electrophoresis in 7.5%, 10%, or 12% SDS-PAGE, transferred onto polyvinylidene fluoride (PVDF) membranes (10600023, Amersham Hybond P 0.45 PVDF, GE Healthcare Life Sciences, Sunderland, UK), and blocked in TBST solution (0.2% Tween 20, 50 mM Tris-HCl pH 7.6, 150 mM NaCl) containing 3% bovine serum albumin or nonfat condensed milk. Membranes were then incubated overnight with the following rabbit primary antibodies: anti-xCT (1:1000; #12691) and anti-phospho-acetyl-CoA carboxylase (pACC, 1:1000; #3661) purchased from Cell Signaling Technology, Danvers, MA, USA), anti-HO-1 (1:1000; ab13243), anti-GCL catalytic subunit (GCLC, 1:1000; ab190685), anti-GCL modifier subunit (GCLM, 1:1000; ab81445), and β-actin (1:2000; ab8227) all purchased from Abcam. After incubation with primary antibodies, membranes were probed with antirabbit HRP-conjugated secondary IgG antibodies (1:4000; ab6721, Abcam). Detection of immunoreactive bands was performed by an enhanced chemiluminescence detection system (sc-2048) (Santa Cruz Biotechnology) using an iBright CL1500 Imaging System (Thermo Fisher Scientific, Carlsbad, CA, USA). Quantitative analysis of immunoreactive bands was conducted densitometrically by ImageJ software (National Institutes of Health) [18]. The ratio of dots per band for the target protein and β-actin (gel loading control) from three independent experiments was averaged, and changes in protein level were expressed as a percentage of an untreated control sample, which was standardized as 100%. 

### 4.6. ELISA Assay for Measurement of TNF-α and IL-6

Sandwich ELISA was used to measure the cytokine content in liver supernatants using MaxiSorp plates (Nunc, Rochild, Denmark). Anti-cytokine-paired antibodies were used for cytokine detection according to the manufacturer’s instructions. The antibodies were: anti-mouse TNF-α purified rabbit monoclonal (clone EPR16803-2), anti-mouse TNF-α biotinylated rabbit monoclonal (EPR16803-84, both from Abcam), anti-mouse IL-6 purified mouse monoclonal (clone MP5-20F3), and anti-mouse IL-6 biotinylated mouse monoclonal (MP5-32011, both from eBioscience). The absorbance was measured using multiplate reader Synergy H1 at 450 nm, with a correction at 690 nm. Standard curves, based on known concentrations of recombinant murine TNF (Abcam) and murine IL-6 (eBioscience) were used for determination of cytokine concentrations in samples and all ELISA test samples were analyzed in duplicates.

### 4.7. Statistical Analyses

Statistical analysis was performed in GraphPad Prism software (GraphPad Software, San Diego, CA, USA). To test data for normality, the Kolmogorov-Smirnov test was used. If the *F* test indicated an overall difference, a one-way analysis of variance (one-way ANOVA) was performed followed by Tukey’s multiple comparisons test. To compare Nrf2 immunopositivity of two lobular zones for each group, Student’s *t* test was performed. The results are presented as mean value ± statistical error (SEM). Statistical significance was set at *p* < 0.05.

## 5. Conclusions

Our study revealed the ferroptotic phenotype of hepatocytes as an important part of the diabetic-induced pathological changes in the liver. Moreover, the results suggest that targeting ferroptosis represents a new, promising approach in the prevention and treatment of commonly observed liver pathologies accompanying diabetes.

## Figures and Tables

**Table 1:** Physical and biochemical characteristics of the experimental groups.

	Ctrl	DM	DM + Fer-1	ANOVA
Body mass (g)	26.8 ± 0.9	23.0 ± 0.8 *	23.3 ± 1.4	F(2) = 4.17 *p* = 0.0300
Blood glucose (mmol/L)	9.0 ± 0.4	16.8 ± 1.7 **	13.9 ± 1.5	F(2) = 7.34 *p* = 0.0043
Serum ALT (U/L)	57.2 ± 5.6	76.2 ± 2.5 *	61.1 ± 5.2 #	F(2) = 4.08 *p* = 0.0472
Serum TG (mmol/L)	1.548 ± 0.02	1.7 ± 0.05 *	1.5 ± 0.05 #	F(2) = 7.78 *p* = 0.0068

Ctrl—control, DM—diabetic, DM + Fer-1—diabetic + Fer-1-treated group. Data are shown as mean ± SEM. Statistical significance: compared with the Ctrl group (*), * *p* < 0.05, ** *p* < 0.01; DM vs. DM + Fer-1 comparison (#), # *p* < 0.05.

## Data Availability

All study data are included in the article and Supplementary Materials.

## Supplementary Materials

The following supporting information can be downloaded at: https://www.mdpi.com/article/10.3390/ijms23169309/s1.
