# Supplementary material for: Involvement of Ferroptosis in Diabetes-Induced Liver Pathology
Source: Int J Mol Sci. 2022 Aug 18;23(16):9309. doi: 10.3390/ijms23169309 (PMC9409200; doi:10.3390/ijms23169309)
Supplement: Supplementary file 1 [file ijms-23-09309-s001.zip › ijms-1828767-SI.pdf]

Original images of Western blot used in Figure 2b, 3b and 5b obtained by iBright CL1500 Imaging System. Protein expression levels of HO-1, xCT, GCLC, GCLM and β-actin in the liver of control (Ctrl), diabetic (DM), and diabetic Fer-1-treated (DM+Fer-1) animals.

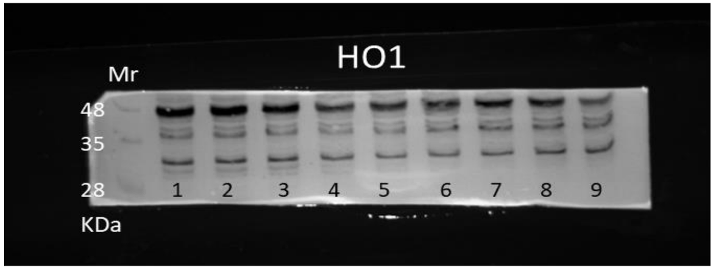

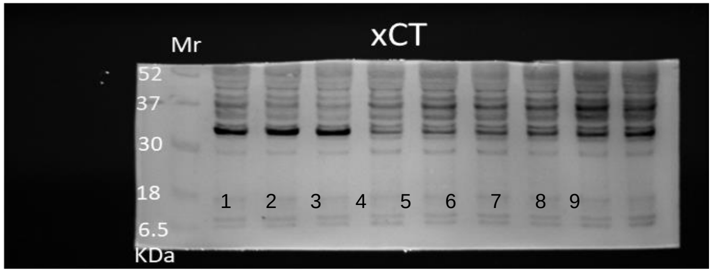

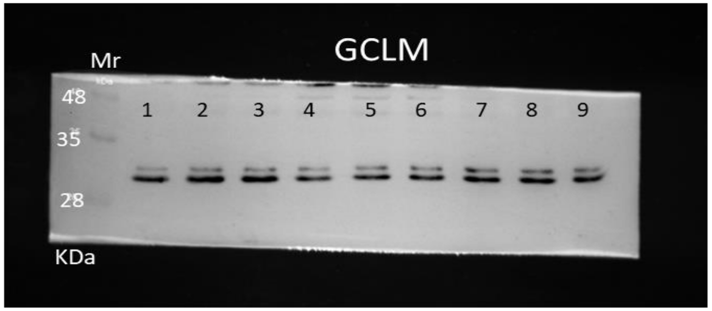

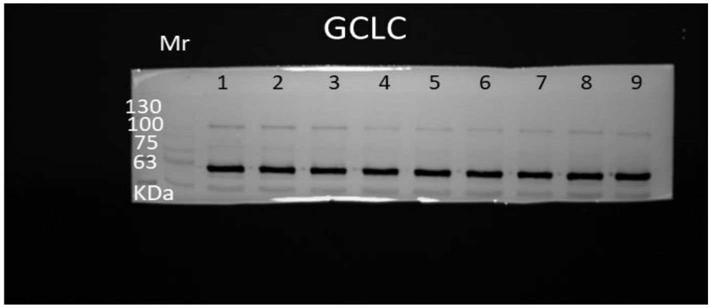

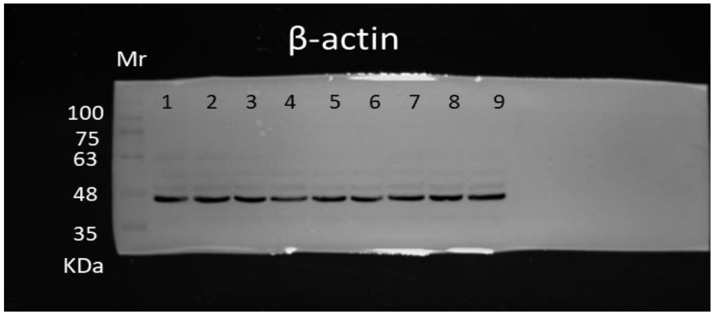

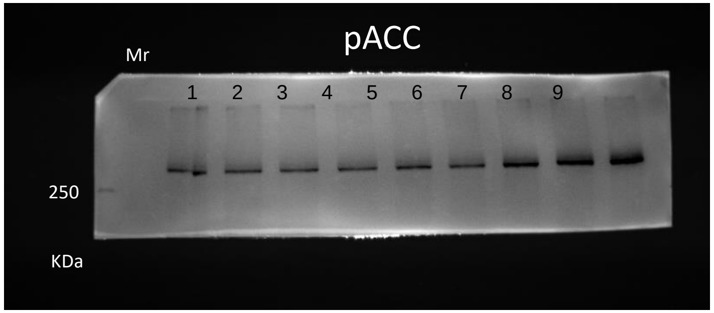

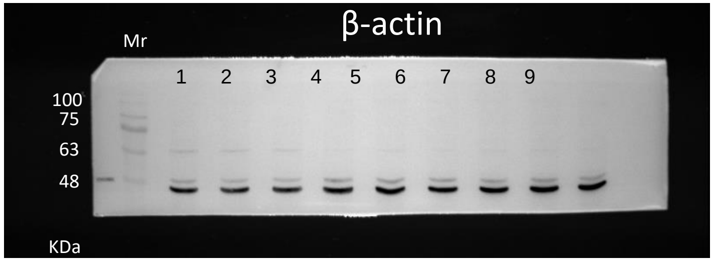

Lines 1,2,3 – Control;

Lines 4,5,6 - DM;

Lines 7,8,9 - DM+Fer-1
